# Supplementary material for: Video-observed therapy (VOT) vs directly observed therapy (DOT) for tuberculosis treatment: A systematic review on adherence, cost of treatment observation, time spent observing treatment and patient satisfaction
Source: PLoS Negl Trop Dis. 2024 Oct 15;18(10):e0012565. doi: 10.1371/journal.pntd.0012565 (PMC11508048; doi:10.1371/journal.pntd.0012565)
Supplement: Attachment — Submitted filename: Rebuttal Letter.docx [file pntd.0012565.s002.docx]

**Rebuttal Letter**

**Dear Editors Dr Poonawala and Dr Pappas,**

We would like to express our sincere gratitude for considering our manuscript titled ***"Video-observed therapy (VOT) vs directly observed therapy (DOT) for tuberculosis treatment: a systematic review on adherence, cost of treatment observation, time spent observing treatment and patient satisfaction"*** for publication in *PLOS Neglected Tropical Diseases*. We also extend our thanks to the reviewers for their thorough evaluations and insightful comments, which have greatly contributed to improving the quality of our work.

After carefully considering each of the reviewers' comments, we have made the necessary revisions to address their concerns. We believe these revisions have significantly enhanced the clarity and robustness of our manuscript.

We chose to submit our work to *PLOS Neglected Tropical Diseases* because we believe it is the ideal platform for disseminating our research. Our study addresses tuberculosis, one of the leading neglected diseases globally, particularly in low- and middle-income countries where treatment adherence challenges are most pronounced. Video-observed therapy (VOT) represents a significant innovation that can transform tuberculosis management in resource-limited settings, aligning perfectly with *PLOS Neglected Tropical Diseases'* mission to advance scientific research in areas that have historically been underfunded and underexplored.

Moreover, we are confident that the readers of *PLOS Neglected Tropical Diseases* are deeply invested in innovative interventions that can improve treatment effectiveness and patient satisfaction in the context of neglected diseases. Our study not only provides comparative evidence between VOT and DOT but also offers practical insights that can influence public health policies on a global scale.

We are convinced that *PLOS Neglected Tropical Diseases* is the best venue to reach an audience committed to improving health outcomes for vulnerable populations. We sincerely hope that our manuscript will be accepted for publication and contribute to advancing knowledge in this critical area.

Once again, we appreciate the opportunity to submit our work to *PLOS Neglected Tropical Diseases* and look forward to your feedback on our submission.

Sincerely,

*Thiago Areas Lisboa Netto
Evandro Chagas National Institute of Infectious Diseases (FIOCRUZ)
Rio de Janeiro, Brazil*

Reviewer #1:

**On l. 93, the authors mentioned that the study was conducted in accordance with PRISMA. A filled-out PRISMA checklist should be available, and the authors should ensure all relevant requirements are fulfilled.**

Response: We apologize for the oversight. We have now included a filled-out PRISMA checklist as an appendix to the manuscript and ensured that all relevant requirements are fulfilled.

Line 108:
A comprehensive database search was conducted in accordance with the Preferred Reporting Items for Systematic Reviews and Meta-Analyses (PRISMA) guidelines. A filled-out PRISMA checklist is provided as an appendix to this manuscript**.** The database search, carried out in July 2023, utilized MEDLINE, Embase, and Cochrane to find studies comparing VOT with DOT. The search terms used included:

**On l. 131, the authors should include a citation for the Cochrane risk-of-bias tool.**

Response: We have included the citation for the Cochrane risk-of-bias tool in the Methods section. The reference has been added as follows:

Line 346:
Higgins JPT, Altman DG, Gøtzsche PC, et al. The Cochrane Collaboration’s tool for assessing risk of bias in randomized trials. BMJ. 2011;343

.

Reviewer #2:

**The methods are simple but meet the stated objectives. It would be useful to further define DOT - for example in some studies DOT was in-person delivered to the home, while in others DOT meant that people had to go somewhere for their medication. This difference likely had a substantial effect on adherence and preference.**

Response: We have now elaborated on the definition of DOT in the Methods section, specifying the variations in DOT implementation across studies.

Line 120:
Directly Observed Therapy (DOT) was defined differently across studies. In some instances, DOT involved in-person delivery of medication to the patient's home, while in other cases, patients were required to travel to a specific location to receive their medication. These variations in DOT implementation likely had a substantial effect on adherence and patient preference.

**It would also be good to have some descriptors of the people included in these studies, age and others that are included across the studies if possible.**

Response: We have added demographic characteristics, such as age and gender distribution, in Table 1 and summarized these details in the Results section.

Line 184:
The study periods ranged from 2014 to 2019, and they were conducted in Moldova, England, China, and the USA. Each study enrolled both male and female participants, with a greater proportion of men compared to women (686 men versus 329 women) and with the age range encompassing individuals aged 16 and above. All studies were randomized controlled trials.

Reviewer #1: Results

**On ll. 139-140, the authors mention the total number of included studies and participants. Before this, it would be appropriate to describe the results of the search and selection process, from the number of records identified in the search to the number of studies included in the review and refer to Figure 1.**

Response: We have revised the Results section to include a description of the search and selection process and referred to Figure 1.

Line 167:
A total of 910 studies were identified, of which 891 were excluded either for being unrelated to the topic of discussion or for being duplicates. Of the remaining 19 studies, 4 met the inclusion criteria and were included in the analysis. In total, 1,044 patients were randomized across these selected studies. These data are summarized in Figure 1. The baseline characteristics of the studies are shown in Table 1, and the summary of the findings from each analysis is detailed in Table 2.

**Table 1 could benefit from including relevant demographic characteristics for the included participants such as summary statistics for age, proportion of male/female participants, etc.**

Response: We have updated Table 1 to include relevant demographic characteristics, including age and gender distribution.

Line 181:

**Ll. 148-151 are perhaps a bit too unspecific. What was the proportion of male/female participants, what were the median or mean ages, what were the specific age ranges?**

Response: We have provided more specific details regarding the proportion of male/female participants, median/mean ages, and specific age ranges in the Results section.

Line 184:
The study periods ranged from 2014 to 2019, and they were conducted in Moldova, England, China, and the USA. Each study enrolled both male and female participants, with a greater proportion of men compared to women (686 men versus 329 women) and with the age range encompassing individuals aged 16 and above. All studies were randomized controlled trials.

**While Table 3 provides a good overview of the quality assessment, a summary of its content should be included in the results section, referring to the table.**

Response: We have included a summary of the quality assessment results in the Results section and referred to Table 3.

Line 210:
The risk of bias in each study using the Cochrane risk-of-bias tool is detailed in Table 3. Overall, the studies exhibited a low risk of selection bias. Performance bias was consistently high across all studies. Detection bias was low in the studies by Story et al. (2019), Burzynski et al. (2020), and Guo et al. (2020), while Ravenscroft et al. (2020) presented an unclear risk. Attrition bias generally showed a low risk except in Story et al. (2019), which had a high risk. Reporting bias was unclear in half of the studies, indicating variability in how outcomes were reported(6, 7, 8, 9).

Reviewer #1: Study limitations

**On ll. 211-214, the authors discuss the implications of treatment observers not being blinded. This part of the discussion could also elaborate on how this potential source of bias might have affected the findings of the review.**

Response: We have elaborated on how the lack of blinding of treatment observers might have introduced performance bias and affected the findings in the Discussion section. Besides that, we have added a paragraph discussing the absence of subgroup statistical analysis of sociodemographic and social determinants, such as age, sex, comorbidities, educational level, history of homelessness and drug or alcohol abuse.

Line 260:
Our study has several limitations. Firstly, the fact that both patients and treatment observers were not blinded to the intervention introduces potential bias. This lack of blinding may have led to performance bias, which could have significantly influenced the behavior of both patients and observers. For instance, patients aware that they were being observed via video might have been more adherent to their treatment regimen than they would have been under unobserved conditions, thus inflating adherence rates. Additionally, this performance bias could impact the interpretation of data related to secondary outcomes, such as patient satisfaction and time spent observing treatment, as the presence of the video interface might have altered the natural dynamics of treatment observation compared to in-person methods. Therefore, the interpretation of the effectiveness of VOT must be approached with caution, taking into account the possibility that the unblinded nature of these studies may have led to overestimated benefits.

Line 275:
It is important to highlight that subgroup statistical analysis of sociodemographic and social determinants, such as age, sex, comorbidities, educational level, history of homelessness and drug or alcohol abuse were not reported by any of the studies included. The absence of this data is significant, as these factors can greatly influence patient adherence and treatment outcomes. For instance, comorbidities and substance abuse may affect both the effectiveness of the treatment and the likelihood of patients adhering to the treatment protocols. The nonexistence of subgroup analysis limits our understanding of how these factors might contribute to disparities in adherence and treatment success. Without this information, it is challenging to identify whether certain groups are disproportionately affected by the interventions studied or if specific barriers are hindering their adherence. This gap in the data could impact the applicability of the study's findings across different patient populations and settings(10).

**The limitations of the review process are not currently discussed. It is important to discuss limitations such as the potential impact of excluding non-English studies or the fact that large databases such as Scopus and Web of Science were omitted from the search.**

Response: We have expanded the Study Limitations section to discuss the potential impact of excluding non-English studies and not searching databases such as Scopus and Web of Science.

Line 304:
Furthermore, our review’s comprehensiveness may have been impacted by the exclusion of non-English studies and the omission of other relevant databases from our search**.** This limitation potentially restricts the inclusion of valuable data from a broader range of sources.

**It would also be beneficial to discuss the role of publication bias and how it may have affected the results of the review.**

Response: We have included a discussion on the role of publication bias and its potential impact on our review results in the Study Limitations section.

Line 307:
Additionally, publication bias could have influenced our results, as studies with positive findings on VOT are more likely to be published and included in the review, which can lead to an overrepresentation of positive results in the literature. Such selective publication practices could distort the true benefits and overall impact of VOT, thereby affecting the conclusions drawn from our analysis.

Reviewer #1: Conclusions

**The conclusion is well-written and offers a concise summary of the main findings and their implications. While it does mention that further research is warranted, it should also include some of the limitations from the discussion.**

Response: We have revised the Conclusion section to include some of the key limitations discussed in the Discussion section.

Line 318:
The results of this systematic review, which included over 1,000 patients, suggest that VOT, compared to traditional DOT, enhances treatment adherence, reduces the burden of treatment observation in terms of cost and time, and increases patient satisfaction. Nevertheless, these findings should be interpreted with caution due to some limitations. These include the lack of blinding in the included studies, the exclusion of drug-resistant tuberculosis and elderly patients, the absence of statistical analysis for subgroups based on sociodemographic characteristics and social determinants, and most significantly, the potential impact of access to technology and the internet on the generalizability of the results, as such access may not be available to all populations. Therefore, further research is warranted to ensure VOT's effective integration into diverse healthcare settings, particularly in low- and middle-income countries (LMICs), where its impact could be substantial.

Reviewer #2:

**One wonders if these studies would be generalizable: for example, VOT requires that people have a device with internet access. Populations in this study are a select group and this might be included in the discussion.**

Response: We have expanded the “Study Limitations” section and included a discussion in the “Conclusions” section on the generalizability of the study findings, emphasizing that VOT requires access to technology and internet, which may limit its applicability in certain populations.

Line 293:
Additionally, VOT may not be universally applicable due to disparities in internet and technology access. This raises concerns about the generalizability of the findings, particularly to areas with limited internet access(11), more likely to be in low- and middle-income countries (LMICs), which hold the greatest burden of both TB and drug-resistant TB globally(12). Specific challenges include inadequate internet infrastructure, high costs of internet access, and limited availability of smartphones or devices capable of supporting video calls. Besides that, there may be limited digital literacy among patients and healthcare workers, which can further complicate the implementation of VOT. The lack of reliable technology infrastructure and digital support could significantly hinder the adoption of VOT. Consequently, the effectiveness and feasibility of VOT in these settings might be compromised, making it challenging to apply the study’s results broadly. However, as internet and smartphone access continue to improve, particularly in LMICs, VOT may become a more viable option for tuberculosis treatment in these regions(13).

Line 318:
The results of this systematic review, which included over 1,000 patients, suggest that VOT, compared to traditional DOT, enhances treatment adherence, reduces the burden of treatment observation in terms of cost and time, and increases patient satisfaction. Nevertheless, these findings should be interpreted with caution due to some limitations. These include the lack of blinding in the included studies, the exclusion of drug-resistant tuberculosis and elderly patients, the absence of statistical analysis for subgroups based on sociodemographic characteristics and social determinants, and most significantly, the potential impact of access to technology and the internet on the generalizability of the results, as such access may not be available to all populations. Therefore, further research is warranted to ensure VOT's effective integration into diverse healthcare settings, particularly in low- and middle-income countries (LMICs), where its impact could be substantial.

Editorial and Data Presentation Modifications:

**In Table 2 in the column "Adherence" on the second row, the text is positioned too far to the left.**

Response: We have corrected the alignment of the text in the "Adherence" column of Table 2.

Line 207:

**Neither Figure 1 nor Table 3 are mentioned in the article. Their content should be summarized under results.**

Response: We have now mentioned and summarized the content of Figure 1 and Table 3 in the Results section.

Line 167:
A total of 910 studies were identified, of which 891 were excluded either for being unrelated to the topic of discussion or for being duplicates. Of the remaining 19 studies, 4 met the inclusion criteria and were included in the analysis. In total, 1,044 patients were randomized across these selected studies. These data are summarized in Figure 1. The baseline characteristics of the studies are shown in Table 1, and the summary of the findings from each analysis is detailed in Table 2.

Line 210:
The risk of bias in each study using the Cochrane risk-of-bias tool is detailed in Table 3. Overall, the studies exhibited a low risk of selection bias. Performance bias was consistently high across all studies. Detection bias was low in the studies by Story et al. (2019), Burzynski et al. (2020), and Guo et al. (2020), while Ravenscroft et al. (2020) presented an unclear risk. Attrition bias generally showed a low risk except in Story et al. (2019), which had a high risk. Reporting bias was unclear in half of the studies, indicating variability in how outcomes were reported(6, 7, 8, 9).

We appreciate the recognition of the strengths of our manuscript and have made the suggested revisions to enhance its clarity and comprehensiveness. We believe these revisions have addressed all the reviewers' comments and improved the overall quality of the manuscript.

We hope that the revised manuscript meets your approval and look forward to your positive response.

Sincerely,

*Thiago Areas Lisboa Netto
Evandro Chagas National Institute of Infectious Diseases (FIOCRUZ)
Rio de Janeiro, Brazil*
